# The facial expression of schizophrenic patients applied with infrared thermal facial image sequence

**DOI:** 10.1186/s12888-017-1387-y

**Published:** 2017-06-24

**Authors:** Bo-Lin Jian, Chieh-Li Chen, Wen-Lin Chu, Min-Wei Huang

**Affiliations:** 1 Department of Aeronautics and Astronautics, National Cheng Kung University, Tainan, 701 Taiwan; 2 Institute of Biomedical Engineering, National Cheng Kung University, Tainan, 701 Taiwan; 3 Department of Psychiatry, Chiayi Branch, Taichung Veterans General Hospital, Chia-Yi, 600 Taiwan

**Keywords:** Affine registration, Emotion assessment, Schizophrenia, Skin temperature, Thermal imaging

## Abstract

**Background:**

Schizophrenia is a neurological disease characterized by alterations to patients’ cognitive functions and emotional expressions. Relevant studies often use magnetic resonance imaging (MRI) of the brain to explore structural differences and responsiveness within brain regions. However, as this technique is expensive and commonly induces claustrophobia, it is frequently refused by patients. Thus, this study used non-contact infrared thermal facial images (ITFIs) to analyze facial temperature changes evoked by different emotions in moderately and markedly ill schizophrenia patients.

**Methods:**

Schizophrenia is an emotion-related disorder, and images eliciting different types of emotions were selected from the international affective picture system (IAPS) and presented to subjects during ITFI collection. ITFIs were aligned using affine registration, and the changes induced by small irregular head movements were corrected. The average temperatures from the forehead, nose, mouth, left cheek, and right cheek were calculated, and continuous temperature changes were used as features. After performing dimensionality reduction and noise removal using the component analysis method, multivariate analysis of variance and the Support Vector Machine (SVM) classification algorithm were used to identify moderately and markedly ill schizophrenia patients.

**Results:**

Analysis of five facial areas indicated significant temperature changes in the forehead and nose upon exposure to various emotional stimuli and in the right cheek upon evocation of high valence low arousal (HVLA) stimuli. The most significant *P*-value (lower than 0.001) was obtained in the forehead area upon evocation of disgust. Finally, when the features of forehead temperature changes in response to low valence high arousal (LVHA) were reduced to 9 using dimensionality reduction and noise removal, the identification rate was as high as 94.3%.

**Conclusions:**

Our results show that features obtained in the forehead, nose, and right cheek significantly differed between moderately and markedly ill schizophrenia patients. We then chose the features that most effectively distinguish between moderately and markedly ill schizophrenia patients using the SVM. These results demonstrate that the ITFI analysis protocol proposed in this study can effectively provide reference information regarding the phase of the disease in patients with schizophrenia.

## Background

Although studies using schizophrenia-specific magnetic resonance imaging (MRI) and electroencephalograms can help in understanding structural differences in the brain and the responsiveness within brain regions, numerous restrictions related to MRI, including high cost and reluctance of subjects to undergo such testing, limit the use of these procedures [1]. Schizophrenia is a degenerative neurological disease, leading to 20% of a patient’s lifetime being spent as years lived with disability (YLDs) [2]. Many studies have been conducted for schizophrenia, which understandably involves a wide range of aspects; however, the results of these studies vary greatly. As heterogeneity among patients is frequently not taken into account by investigators when analyzing data, the features specific to certain variant phases of the disease often remain undetected, hindering an in-depth understanding of the symptoms. In this study, we used a digital infrared thermal image system (DITIS) to image the subjects and measure their temperatures without contact, which enabled us to conduct an in-depth analysis and quantification of the thermal state upon evocation of emotions in schizophrenia patients with reduced heterogeneity.

Since each person’s subjective judgment is influenced by psychological and physiological factors, and subjective factors can alter psychological and physiological responses and influence the evaluation of emotion, studies addressing emotions often use non-subjective methods to evoke emotional responses. Inner emotion and cognitive activity can be reflected through facial expressions, behavioral responses, sound, etc. Facial expression represents a type of non-verbal interaction; thus, in recent years, many studies have explored the identification of facial expression under visible light [3, 4]. However, these studies use a common blind spot, in which non-spontaneous expression of emotions can be camouflaged, resulting in misjudgment of emotions [5]. In addition to this blind spot, recognition of facial expression is also influenced by environmental illumination and face poses, leading to system identification errors [6]. Therefore, many studies have started to use infrared thermal facial images (ITFIs) technology, as it reduces the impact of lighting and offers the advantage of a non-contact approach, which is suitable for human psychological and physiological studies [7–12]. Moreover, it has been shown that many health and emotional states are associated with variations in facial temperature [13–17]. During ITFI collection, subjects’ heads are not stabilized in order to make them feel as comfortable and natural as possible, but this can cause difficulties for subsequent analyses. Therefore, the calibration and alignment of images become very important. Image calibration can be performed using two methods: one based on features and the other on areas. Image-based calibration is not suitable for situations with significant nonlinear differences [18]. Moreover, the characteristics of infrared thermal images are different from those of images taken under visible light [19].

In summary, we used ITFI to investigate changes in the variation of the temperature in each facial area upon visual stimulation-evoked emotions. Based on our results, we were able to successfully differentiate between moderately and markedly ill schizophrenia patients.

## Methods

### Participants

The PANSS test is commonly applied in the clinical identification of schizophrenia. In this study, we used the total scores of PANSS to classify all participants into moderately ill and markedly ill groups [20, 21]. There were 18 patients in the moderately ill group (mean age: 42.61 ± 7.16 years; age of illness onset: 26.8 ± 8.75 years old; illness duration: 17.56 ± 6.65 years; 8 men, 10 women; medication (e.g., chlorpromazine equivalent, mg): 183.33 ± 103.41 mg) and 17 patients in the markedly ill group(mean age: 42.47 ± 7.67 years; age of illness onset: 24.59 ± 6.69 years old; illness duration: 18.53 ± 7.32 years; 9 men, 8 women; medication (e.g., chlorpromazine equivalent, mg): 223.35 ± 100.22 mg) as shown in Table 1. Demographic data such as age, sex, age of illness onset, illness duration and medication were not different between the two groups.

**Table 1:** Comparison of demographic characteristics between moderately ill and markedly ill schizophrenia patients

Demographic variables	Moderately ill (*n* = 18)	Markedly ill (*n* = 17)	*P* value
Age (Years)	42.61 ± 7.16	42.47 ± 7.67	0.940
Sex (Male/Female)	8/10	9/8	0.627
Age of illness onset (y/o)	26.5 ± 8.75	24.59 ± 6.69	0.475
Illness duration (years)	17.56 ± 6.65	18.53 ± 7.32	0.683
Medication (e.g., chlorpromazine equivalent, mg)	183.33 ± 103.41	222.35 ± 100.22	0.266
PANSS scores			
Total	75.06 ± 14.65	100.65 ± 11.66	0.000
Positive	17.22 ± 3.49	27.76 ± 3.60	0.000
Negative	19.78 ± 5.29	24 ± 3.88	0.011
Global	38.06 ± 8.52	48.88 ± 6.52	0.000

The participants were recruited from the outpatient clinic at the Department of Psychiatry, Chiayi and Wanqiao Branch, Taichung Veterans General Hospital, Chiayi, Taiwan. Participants underwent screening that included their medical and psychiatric histories, laboratory test results, an illicit drug screening, and a physical examination. A psychiatric diagnosis of schizophrenia was established using the structured clinical interview from the DSM-IV and a semi-structured interview conducted by a research psychiatrist [22]. After receiving a complete explanation of the study procedures, all participants provided written informed consent as approved by the institutional review board. This study was approved by the ethics committee of the Taichung Veterans General Hospital, and was conducted in accordance with Good Clinical Practice procedures and the current revision of the Declaration of Helsinki [23].

### Stimuli and paradigm

**Fig. 1:**
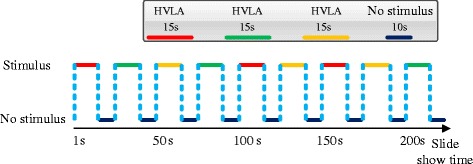
Schematic diagram of the relationship between the time and the order of the images. During each test, each picture was displayed for 3 s, and patients were allowed 10 s of rest after one stimulation unit that consisted of five pictures of the same type. The three types of emotion appeared three times over the course of the entire 225 s test time

Lang et al. recorded the sensitivity of subjects to image stimuli, and objectively established the visual complexity and emotional response rating criteria [24]. They built an emotional stimulus database called the International Affective Picture System (IAPS), which include more than 1000 color images (see http://csea.phhp.ufl.edu/Media.html). Similar to several previous studies IAPS has clear content, test-retest reliability, and contains many different categories of pictures such as human emotions (e.g., happy, sad, disgust and angry) with different figure photo types and articles [25–27]. Using IAPS images, subjects can induce personal emotional experiences and reactions. Many studies use IAPS as visual stimuli to evaluate emotional and electrophysiological responses. We have also used IAPS images to evoke different emotions in subjects. We selected 45 images for a questionnaire survey to be filled out by 100 normal individuals (48 males with a mean age of 35.94 ± 12.38 and 52 females with a mean age of 37.45 ± 14.14) who were born and raised in Taiwan. These emotional pictures were divided into valence and arousal with dimensions, and the nine-point Likert scale was used for analysis. The results divided into three different types of emotion called HVLA (High Valence Low Arousal; valence: 7.42 ± 0.51 arousal: 4.77 ± 0.37), LVLA (Low Valence Low Arousal; valence: 3.26 ± 0.53 arousal: 4.55 ± 0.86) and LVHA. (Low Valence High Arousal; valence: 1.21 ± 0.59 arousal: 6.45 ± 0.56). The film was split into fragments separated by breaks. The film lasted 225 s in total, and there were 9 breaks lasting 10 s each. Each segment evoked 3 types of emotions and lasted 15 s, and there were 3 segments for each emotions type. Each segment consisted of 5 images, and each image was presented for 3 s. The order and the duration of image presentation is shown in Fig. 1.

### Thermal image data acquisition

A digital infrared thermal image system (DITIS) (Spectrum 9000-MB Series; United Integrated Service Co. Ltd.) was used to collect images. The collected ITFI were 320 × 240 temperature matrix data, and the sampling rate was 2 frames per second (fps). During data collection, access to the experimental venue was prevented in order to avoid strong environmental convection and to maintain the ambient temperature between 26 °C and 28 °C. The interior of the room was covered by three layers of shades to minimize heat radiation. To ensure the rights and safety of the subjects and under the principle of comfort and minimal harm, the head was not stabilized during the experimental process.

### Image registration processing

**Fig. 2:**
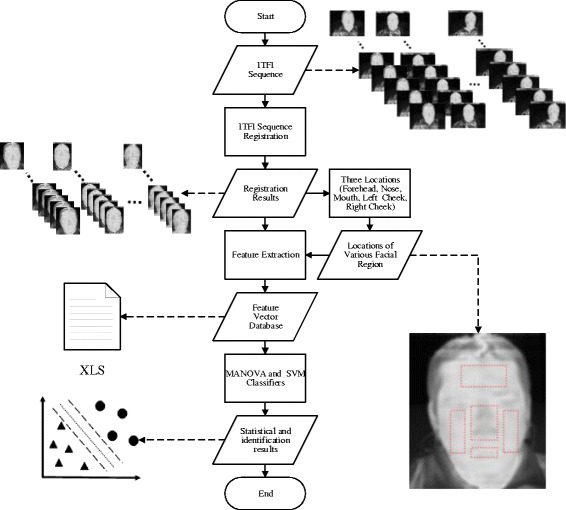
Experimental procedure. First, ITFIs are collected and calibrated. The calibration process can minimize the variation of facial images caused by movements. Then, each person’s forehead, nose, mouth, left cheek, and right cheek areas are selected. The average skin temperature in each area is calculated. For features extraction, average temperatures are obtained according to the time sequence of visual stimulation generated by emotion pictures. MANOVA and SVM classifier are used to analyze and identify moderately and markedly ill schizophrenia patients

The presentation of IAPS images to the subjects was performed according to previously established order and duration, and the ITFI were collected simultaneously with the presentation. The subsequent procedures are shown in Fig. 2. To ensure the comfort of and minimal harm to the subjects, their head was not fixed during the collection of experimental data, which led to slight shifts of ITFIs over time and was unfavorable for subsequent analysis. Thus, affine registration processing technology was used to reduce the variation of sequential images in order to ensure the appropriate overlapping of facial image locations. This process negates the influence of facial movements on the validity of facial area correlation analyses. For calibration, center-of-mass localization of the binocular area, image translation, and flip were mainly used to generate fixed images for the calibration. Then, the two-stage genetic algorithm (GA) was used to automatically complete affine registration of ITFIs. This method effectively improved the overlap error before and after image calibration, effectively compensating for spontaneous, small, unconscious movements of the human face [28].

### Features extraction and analysis

From calibrated thermal sequential images of each subject, images of five areas including the forehead, nose, mouth, left cheek, and right cheek were selected for analysis, and the average temperature of each area was calculated. Since the test lasted for 225 s and the FPS of the DITIS was 2, there were a total of 450 features per facial area. Based on the stimulation time for each type of emotion, 48 features per type (HVLA, LVLA, and LVHA) were established. After reducing data dimensions using principal component analysis (PCA), nine features per type of emotion were obtained, and less important information was filtered out [29]. Multivariate analysis of variance (MANOVA) was used for statistical analysis, while a support vector machine (SVM) was used as a classifier to train and identify the results. Well-known identification algorithms included neural network (NN), SVM, learning vector quantization (LVQ), and other intelligent classifiers. To address the issues related to multi-class identification and quickly establish the available identifier structure in addition to verifying the feasibility of the procedure, this study used the “classification learner” application of Matrix Laboratory (MATLAB) R2015b (MathWorks, USA) to obtain SVM training and classification.

## Results

### MANOVA of facial areas in response to evoked emotions in moderately and markedly ill schizophrenia patients

According to the experimental procedure shown in Fig. 2, *P*-values obtained from the forehead, nose, mouth, left cheek, and right cheek following evocation of HVLA, LVLA, and LVHA in moderately and markedly ill schizophrenia patients were analyzed. The *p*-value for the MANOVA was calculated using Wilks’ Lambda. The groups of moderately and markedly ill schizophrenia patients were considered independent variables, while the features of emotions corresponding to their facial areas were taken as dependent variables, as shown in Table 2. The results indicated that the difference in the average temperature changes in the forehead area between moderately and markedly ill schizophrenia patients was the most significant. Upon evocation of three types of emotion in this area, the differences were all highly significant, including the *P*-value under evoked LVHA, which was lower than 0.001and represented the highest level of significance. In addition, the differences in the nose area under the three types were all significant, especially under evoked LVLA where the *P*-value was most significant at0.002. In addition, the *P*-value obtained for the right cheek area under evoked HVLA was 0.018.

**Table 2:** MANOVA results for each facial area under three evoked emotions in moderately and markedly ill schizophrenia patients

			Emotion types (number of features)			
			HVLA (9)	LVLA (9)	LVHA (9)	No Stimulus (9)
Facial region	Forehead	F-value	4.751	4.638	7.303	4.741
		*P*-value	0.001**	0.001**	0.000***	0.001**
	Nose	F-value	3.201	4.101	2.457	2.670
		*P*-value	0.01*	0.002**	0.037*	0.025*
	Mouth	F-value	1.324	1.247	0.709	1.349
		*P*-value	0.274	0.312	0.613	0.263
	Left Cheek	F-value	1.526	2.126	1.038	1.321
		*P*-value	0.191	0.066	0.437	0.276
	Right Cheek	F-value	2.865	0.980	0.980	2.583
		*P*-value	0.018*	0.479	0.479	0.087

According to the variable for the feature and factors in the fixation of group

*P*-value <0.05* *P*-value <0.01** *P*-value <0.001***

“F” is F-value. “*P*-value” is significance

### SVM identification results using different numbers of features

The difference in average temperature changes between the moderately and markedly ill schizophrenia patients was the most significant in the forehead area under evoked LVHA (Table 2). Thus, when the 48 features were reduced to 8–10 using dimensional reduction by PCA, identification was performed by linear, quadratic, cubic, and medium Gaussian kernels of the SVM classifier using different numbers of features. The results are shown in Table 3. The highest identification rates for 9 features were obtained using quadratic and medium Gaussian kernels (up to 94.3%), followed by linear and cubic kernels (91.4%). The identification rates of the remaining features ranged from 77.1 to 91.4%, and the highest average rate was obtained when the number of features was 9, indicating the greatest difference between moderately and markedly ill schizophrenia patients in this context.

**Table 3:** SVM identification results using different numbers of features

Classifier	Kernel	LVHA (8)	LVHA (9)	LVHA (10)	LVHA (11)	LVHA (12)
SVM	Linear	91.4	91.4	91.4	88.6	82.9
	Quadratic	88.6	94.3	88.6	80	80
	Cubic	82.9	91.4	85.7	82.9	77.1
	Medium Gaussian	91.4	94.3	88.6	80	88.6

## Discussion

Many studies conducted on thermal imaging measure the temperature response in the orbit as an index [30–33], but such measurements are inconvenient for subjects with myopia who need to wear glasses. Forehead measurements overcome such restrictions, in addition to those arising when subjects wearing contact lenses are reluctant to undergo the orbit test. In a study by Colin et al., an association between the forehead and emotions was observed [34]. Zhu et al. confirmed these findings and used thermal imaging of the forehead as a polygraph with a success rate of 76.3% [35]. In addition, studies on psychological stress demonstrated that temperature changes in the nose were highly correlated with stress [36, 37]. Using thermal imaging, Ioannou et al. also found that temperature changes in the nose area were associated with emotions [38]. These results are consistent with the statistically significant changes in the forehead and nose observed in this study. They showed that under evoked HVLA, LVLA, and HVLA, average temperature changes in the forehead and nose reflect the differences between moderately and markedly ill schizophrenia patients. The difference in average temperature change in the right cheek upon the evocation of HVLA was also significant.

Studies have addressed the effect of frontal brain asymmetry on emotional reactivity. The left prefrontal lobe has been associated with hyperarousability and positive emotions, while the right prefrontal lobe has been related to emotion avoidance and stronger negative emotions [39, 40]. The results in Table 2 show an important difference in the right cheek temperature variation between moderately and markedly ill schizophrenia patients following exposure to HVLA stimuli. Positive emotion induced a strong response in the left prefrontal cortex, effectively reflecting the differences in the right cheek. The results of this study are consistent with the theory of frontal brain asymmetry and emotional reactivity. In addition, Table 2 shows the features in the forehead and nose obtained during rest for which the differences were also statistically significant. We believe that these features may reflect temperature changes that occur after emotional stimulation. If more pictures (emotional stimuli) had been used, the statistical results for the no stimulus values may have been more significant. As this result was consistent with the statistical results from the forehead, the results obtained from rest values were used as a baseline standard and were not included in the investigation on emotional responses in moderately and markedly ill schizophrenia patients.

Dimension reduction using the PCA method [29] was employed to reduce the number of features under evoked LVHA, and different kernels in the SVM classifier were then used to identify moderately and markedly ill schizophrenia patients. The results showed that the recognition rate of quadratic and medium Gaussian kernels for 9 features was 94.3%, which also revalidated the MANOVA results presented in Table 2. The average temperature change in the forehead area under evoked LVHA may represent a distinguishing feature between moderately and markedly ill schizophrenia patients. This result is very exciting. Using the analysis model presented in this study and a sufficiently large database, a reliable mathematical model could be generated. In the future, data could be collected as described in this study and entered into a model to predict a subject’s condition, which could be used as a reference for the evaluation of the disease state. In addition, combined with the Diagnostic and Statistical Manual of Mental Disorders 4th edition (DSM-IV) and a semi-structured interview conducted by a research psychiatrist, the described method may also be used as an adjuvant identification method for moderately and markedly ill patients. Moreover, this study also provides a reference standard for the choice of classifiers and features.

## Conclusions

Under the emotions evoked by visual stimulation, the ITFIs were divided into five areas, the average temperature within each area was calculated, and the continuous average temperature change was used as a feature. Results obtained using MANOVA show that responses in the forehead, nose, and right cheek area were significantly different between moderately and markedly ill schizophrenia patients. Such a difference in the right cheek is consistent with brain asymmetry and emotional reactivity theory. Finally, we found that the features generated by the largest difference in responses to emotions can effectively identify moderately and markedly ill schizophrenia patients using an SVM. These results demonstrate that the analysis of ITFI described in this study can effectively provide clinical reference information on the disease phase in schizophrenia patients.

## Abbreviations

DITIS: Digital infrared thermal image system
HVLA: High valence low arousal
IAPS: International Affective Picture System
ITFI: Infrared thermal facial images
LVHA: Low valence high arousal
LVLA: Low valence low arousal
MANOVA: Multivariate analysis of variance
PCA: Principal component analysis
SVM: Support vector machine

## References

[1] Hu Z, Yang W, Liu H, Wang K, Bao C, Song T (2014). From PET/CT to PET/MRI: advances in instrumentation and clinical applications. Mol Pharm.

[2] Vos T, Flaxman AD, Naghavi M, Lozano R, Michaud C, Ezzati M (2012). Years lived with disability (YLDs) for 1160 sequelae of 289 diseases and injuries 1990–2010: a systematic analysis for the global burden of disease study 2010. Lancet.

[3] Beaudry O, Roy-Charland A, Perron M, Cormier I, Tapp R (2014). Featural processing in recognition of emotional facial expressions. Cogn Emot.

[4] Wang ZF, Miao ZJ, Wu QMJ, Wan YL, Tang Z (2014). Low-resolution face recognition: a review. Vis Comput.

[5] Ulukaya S, Erdem CE: Gaussian mixture model based estimation of the neutral face shape for emotion recognition. Digital Signal Processing 2014, 32(0):11–23.

[6] Santhanaganesh AS, Rajakumar PS: Facial expression recognition in various illuminous environment. Digital Image Processing 2014, 6(3).

[7] Ioannou S, Gallese V, Merla A (2014). Thermal infrared imaging in psychophysiology: potentialities and limits. Psychophysiology.

[8] Ioannou S, Morris P, Mercer H, Baker M, Gallese V, Reddy V (2014). Proximity and gaze influences facial temperature: a thermal infrared imaging study. Frontiers Psychology.

[9] Di Giacinto A, Brunetti M, Sepede G, Ferretti A, Merla A: Thermal signature of fear conditioning in mild post traumatic stress disorder. Neuroscience 2014, 266(0):216–223. 10.1016/j.neuroscience.2014.02.009 24561216

[10] Manini B, Cardone D, Ebisch SJ, Bafunno D, Aureli T, Merla A (2013). Mom feels what her child feels: thermal signatures of vicarious autonomic response while watching children in a stressful situation. Front Hum Neurosci.

[11] Rajoub BA, Zwiggelaar R (2014). Thermal facial analysis for deception detection. IEEE Transactions on Information Forensics and Security.

[12] Esposito G, Nakazawa J, Ogawa S, Stival R, Putnick DL, Bornstein MH (2015). Using infrared thermography to assess emotional responses to infants. Early Child Dev Care.

[13] Nhan BR, Chau T (2010). Classifying affective states using thermal infrared imaging of the human face. IEEE Trans Biomed Eng.

[14] Akio N, Munecazu T: Correlation analysis on alpha attenuation and nasal skin temperature. Journal of Statistical Mechanics: Theory and Experiment 2009, 2009(01):P01007.

[15] Levine JA, Pavlidis I, Cooper M (2001). The face of fear. Lancet.

[16] Hahn AC, Whitehead RD, Albrecht M, Lefevre CE, Perrett DI (2012). Hot or not? Thermal reactions to social contact. Biol Lett.

[17] Clay-Warner J, Robinson DT (2015). Infrared thermography as a measure of emotion response. Emot Rev.

[18] Tsai CL, Li CY, Yang G (2010). Lin KS: : the edge-driven dual-bootstrap iterative closest point algorithm for registration of multimodal fluorescein angiogram sequence. IEEE Trans Med Imaging.

[19] Ma J, Zhao J, Ma Y, Tian J (2015). Non-rigid visible and infrared face registration via regularized Gaussian fields criterion. Pattern Recogn.

[20] Furukawa TA, Levine SZ, Tanaka S, Goldberg Y, Samara M, Davis JM (2015). Initial severity of schizophrenia and efficacy of antipsychotics: participant-level meta-analysis of 6 placebo-controlled studies. JAMA psychiatry.

[21] Leucht S, Kane JM, Kissling W, Hamann J, Etschel E, Engel RR (2005). What does the PANSS mean?. Schizophr Res.

[22] Chen Y-T, Huang M-W, Hung I-C, Lane H-Y, Hou C-J (2014). Right and left amygdalae activation in patients with major depression receiving antidepressant treatment, as revealed by fMRI. Behav Brain Funct.

[23] World Medical Association Declaration of Helsinki: ethical principles for medical research involving human subjects. *Jama* 2013, 310(20):2191–2194. 10.1001/jama.2013.281053 24141714

[24] Lang PJ, Bradley MM, Cuthbert BN, Lang PJ, Simons RF, Balaban M. Motivated attention: affect, activation, and action. Attention and orienting: Sensory and motivational processes. 1997:97–135.

[25] Guntekin B, Başar E: A review of brain oscillations in perception of faces and emotional pictures. Neuropsychologia 2014, 58(0):33–51. 10.1016/j.neuropsychologia.2014.03.014 24709570

[26] Jerram M, Lee A, Negreira A, Gansler D (2014). The neural correlates of the dominance dimension of emotion. Psychiatry Res Neuroimaging.

[27] Kukolja D, Popović S, Horvat M, Kovac B, Cosic K (2014). Comparative analysis of emotion estimation methods based on physiological measurements for real-time applications. International Journal of Human-Computer Studies.

[28] Chen C-L, Jian B-L (2015). Infrared thermal facial image sequence registration analysis and verification. Infrared Phys Technol.

[29] Vidal R, Ma Y, Sastry SS: Principal component analysis. In: Generalized Principal Component Analysis. edn. New York, NY: Springer New York; 2016: 25–62.

[30] Perry A, Aviezer H, Goldstein P, Palgi S, Klein E, Shamay-Tsoory SG (2013). Face or body? Oxytocin improves perception of emotions from facial expressions in incongruent emotional body context. Psychoneuroendocrinology.

[31] Lischke A, Berger C, Prehn K, Heinrichs M, Herpertz SC, Domes G (2012). Intranasal oxytocin enhances emotion recognition from dynamic facial expressions and leaves eye-gaze unaffected. Psychoneuroendocrinology.

[32] Pollina DA, Dollins AB, Senter SM, Brown TE, Pavlidis I, Levine JA, et al. Facial skin surface temperature changes during a “concealed information” tes**t**. Ann Biomed Eng. 2006;34(7):1182–9. 10.1007/s10439-006-9143-3 16786391

[33] Shastri D, Merla A, Tsiamyrtzis P, Pavlidis I (2009). Imaging facial signs of neurophysiological responses. IEEE Trans Biomed Eng.

[34] Puri C, Olson L, Pavlidis I. Levine J. Starren J: StressCam. 2005;1725

[35] Zhu Z, Tsiamyrtzis P, Pavlidis I: Forehead Thermal Signature extraction in lie detection. In: 2007 29th Annual International Conference of the IEEE Engineering in Medicine and Biology Society: 22–26 Aug. 2007 2007; 2007: 243–246. 10.1109/IEMBS.2007.4352269 18001935

[36] Engert V, Merla A, Grant JA, Cardone D, Tusche A, Singer T (2014). Exploring the use of thermal infrared imaging in human stress research. PLoS One.

[37] Shastri D, Papadakis M, Tsiamyrtzis P, Bass B, Pavlidis I (2012). Perinasal imaging of physiological stress and its affective potential. IEEE Trans Affect Comput.

[38] Ioannou S, Ebisch S, Aureli T, Bafunno D, Ioannides HA, Cardone D (2013). The autonomic signature of guilt in children: a thermal infrared imaging study. PLoS One.

[39] Davidson RA, Fedio P, Smith BD, Aureille E, Martin A (1992). Lateralized mediation of arousal and habituation: differential bilateral electrodermal activity in unilateral temporal lobectomy patients. Neuropsychologia.

[40] Wheeler RE, Davidson RJ, Tomarken AJ (1993). Frontal brain asymmetry and emotional reactivity - a biological substrate of affective style. Psychophysiology.

